# Detecting the oxidative reactivity of nanoparticles: a new protocol for reducing artifacts

**DOI:** 10.1007/s11051-014-2493-0

**Published:** 2014-06-28

**Authors:** Jiayuan Zhao, Michael Riediker

**Affiliations:** 1 Institute for Work and Health, University of Lausanne, Rte de la Corniche 2, 1066 Epalinges – Lausanne, Switzerland; 2 SAFENANO, Institute of Occupational Medicine, Singapore, 048622 Singapore

**Keywords:** Reactivity, Reactive oxygen species (ROS), Nanoparticles (NPs), Fluorescence, 2′7-dichlorodihydrofluorescein (DCFH), Nanotoxicity, Health and environmental effects

## Abstract

Understanding the oxidative reactivity of nanoparticles (NPs; <100 nm) could substantially contribute to explaining their toxicity. We attempted to refine the use of 2′7-dichlorodihydrofluorescein (DCFH) to characterize NP generation of reactive oxygen species (ROS). Several fluorescent probes have been applied to testing oxidative reactivity, but despite DCFH being one of the most popular for the detection of ROS, when it has been applied to NPs there have been an unexplainably wide variability in results. Without a uniform methodology, validating even robust results is impossible. This study, therefore, identified sources of conflicting results and investigated ways of reducing occurrence of artificial results. Existing techniques were tested and combined (using their most desirable features) to form a more reliable method for the measurement of NP reactivity in aqueous dispersions. We also investigated suitable sample ranges necessary to determine generation of ROS. Specifically, ultrafiltration and time-resolved scan absorbance spectra were used to study possible optical interference when using high sample concentrations. Robust results were achieved at a 5 µM DCFH working solution with 0.5 unit/mL horseradish peroxidase (HRP) dissolved in ethanol. Sonication in DCFH-HRP working solution provided more stable data with a relatively clean background. Optimal particle concentration depends on the type of NP and in general was in the µg/mL range. Major reasons for previously reported conflicting results due to interference were different experimental approaches and NP sample concentrations. The protocol presented here could form the basis of a standardized method for applying DCFH to detect generation of ROS by NPs.

**Electronic supplementary material:**

The online version of this article (doi:10.1007/s11051-014-2493-0) contains supplementary material, which is available to authorized users.

## Introduction

The field of nanotoxicology has emerged alongside the realization of that there has been a great increase in exposure to nanoparticles (NPs; particles <100 nm) from anthropogenic sources, such as power plants and metal fumes (Oberdorster et al. 2005). Because of their small size, the inhalation of NPs can effectively deposit them in the respiratory tract and the alveolar region. Moreover, a small portion can translocate and reach sensitive organs such as the heart (Oberdorster et al. 2005), or even pass through cell membranes (Seaton and Donaldson 2005). Other important size-related features of NPs (and their aggregates and agglomerates) are their proportionately large surface areas (per unit of mass), the much larger number of particles per unit mass and varied associated new physical and chemical properties (Sager et al. 2007; Nichols et al. 2002). These features further increase the biological activity of NPs (Donaldson et al. 2001). Nel et al. (2006) summarized the observed health effects brought about by exposure to NPs and the possible pathophysiological consequences (Nel et al. 2006). The generation of reactive oxygen species (ROS), oxidative stress, and inflammation have been put forward as important potential health effects due to exposure to natural and manufactured NPs (Nel et al. 2006; Brook et al. 2010). These three effects are interlinked, as the generation of ROS will increase oxidative stress, provoking inflammation which can in turn increase levels of ROS (Stone et al. 1998; Brown et al. 2001; Koike and Kobayashi 2006).

ROS are defined as common oxygen-centered or oxygen-related ions, molecules, and radicals. Understanding particle reactivity is a key step toward understanding the toxicology of particulate matter because the generation of ROS it is not the only direct outcome, but could also trigger other effects when particles are inhaled. Too much oxidative stress caused by an imbalance of oxidants and antioxidants can trigger many diseases, such as cardiovascular diseases (Brook et al. 2010). The health effects of particular NPs can be influenced by both their ability to generate ROS themselves and/or the amount of ROS that can attach to those particles. Interestingly, studies using both cellular and acellular approaches have demonstrated that nano-scale particles have a higher oxidative potential than bigger particles; it is suspected that this is due to their larger surface area providing a larger interface for redox reactions (Stone et al. 1998; Wilson et al. 2002; Koike and Kobayashi 2006). The increasing risks of exposure to NPs, and the important role which their generation of ROS has on their toxicity, have made the study of the capacity for NPs to generate ROS essential to the field of nanotoxicology.

Different methods have been used to characterize the generation of ROS by NPs (Foucaud et al. 2007; Pal et al. 2012; Sauvain et al. 2012). Sauvain et al. (2012) compared three acellular tests for assessing NP reactivity, and their results confirmed that different approaches involving different mechanisms exhibit various sensitivities (Sauvain et al. 2012).

Of these three analytical approaches, the 2′7-dichlorodihydrofluorescein (DCFH) assay, developed more than 40 years ago (Chen et al. 2010), currently is one of the most commonly used (Wardman 2007). It has been applied in many studies (Hung and Wang 2001; Foucaud et al. 2007; Zhao and Hopke 2011) and has been valuable for assessing the capacity to generate ROS. Venkatachari and Hopke (2008) subjected several ROS-surrogate compounds from different functional groups to three separate fluorescent probes, namely DCFH, dithiothreitol (DTT), and p-hydroxyphenylacetic acid; they found DCFH was more non-specific to ROS than the other two (Venkatachari and Hopke 2008). The continuously increasing preference for using DCFH as a probe is probably due to the fact that it can be oxidized non-discriminatorily by many ROS functional groups (Chen et al. 2010). It has been one of the most widely used probes for characterizing H_2_O_2_ quantitatively (Black and Brandt 1974), as well as being responsive to other members of the hydroperoxide group, such as tert-butyl hydroperoxide (Venkatachari and Hopke 2008). In the DCFH test, H_2_O_2_ generates a stable linear calibration curve. Previous studies (Hung and Wang 2001; Zhao and Hopke 2011; Venkatachari et al. 2005) also chose H_2_O_2_ as the standard to express levels of ROS since it is not feasible to express all different ROS concentrations one by one. DCFH can also be oxidized by hydroxyl (·OH) and peroxynitrite (ONOO¯) (Crow 1997). Surrogate compounds from organic peroxide, alkyl peroxide radicals, and hypochlorite were also found to respond to DCFH (Venkatachari and Hopke 2008).

For acellular DCFH measurement, we proceed from stable DCFH_2_-DA via initial deacetylation by the addition of a strong alkaline, usually NaOH (Keston and Brandt 1965). DCFH_2_ is then oxidized to DCF through two consecutive single-electron oxidation processes. Firstly, DCFH_2_ loses one electron to become the obligatory intermediate, DCFH; next DCFH loses a further electron to become DCF. DCF can then be transformed into its excited state of DCF* by photo-excitation (Marchesi et al. 1999).

Some groups have nevertheless questioned the value of DCFH as a fluorescent reactant, and have suggested caution because of its unstable nature: it is sensitive to both light and oxygen (Rota et al. 1999; Pal et al. 2012). Moreover, there has never been a uniform approach to using DCFH. Different experimental handling processes and measurement conditions along the steps of a protocol may understandably culminate in different results. For example, both Sager et al. (2007) and Pal et al. (2012) evaluated different methods for dispersing NP samples and demonstrated very different results using different sonication protocols (Sager et al. 2007; Pal et al. 2012). Some previous research has even applied the same NPs to the DCFH method, yet has reported conflicting or even opposite conclusions (Pal et al. 2012; Sauvain et al. 2012). Thus, questions have been asked about whether DCFH can truly be used as a reliable detection method for ROS generated by NPs, putting in doubt the preference that many researchers have given to this probe. Although informative analyses have been performed on certain NPs, conflicting results have served to confuse later researchers who wonder which study is be believed; they have stopped data from being correctly interpreted and used. This can significantly devalue the importance of previous studies and block the progress of future ones.

Such doubts about the reliability of adapting the popular DCFH method to the detection of ROS generated by NPs indicated a need to evaluate and confirm which of the currently used approaches were indeed suitable for NPs and, if any, to create a standardized protocol. Furthermore, the possibility of artifacts when using the DCFH method with NPs would have to be explored and then avoided in order to improve the method’s usability. In order to achieve all this, we evaluated the performance of the DCFH cell-free oxidative reactivity assay using a range of different fundamental set-ups and inputs. This included varying the concentrations of the catalyst and the different chemical reactants, as well as storing the working solution for different lengths of time. We also compared different dispersion media in order to get closer to the best methodological approach for handling real NP samples. Moreover, to best characterize the potential for NPs to generate ROS, we evaluated a range of sample concentrations in order to find which one most accurately expressed reactivity and minimized possible interference with the test. As our findings demonstrate, we have explored the key issues for conducting a successful DCFH analysis of the ROS generated by NPs; they confirm a DCFH protocol with a promising future in nanotoxicological studies. Moreover, our evaluation of earlier DCFH methods provided some insightful information on which approaches should be preferred for the study of NPs; it helps to explain why there were disagreements over previously published results and to weigh up which results should be trusted.

## Experimental section

### Preparation of the fluorescent probe and standard

This study used DCFH as the fluorescent probe. A stock solution of the probe was made by dissolving DCFH-DA powder into an alcohol reagent. The deacetylation of DCFH-DA was carried out by adding a strong base of sodium hydroxide (NaOH). This solution was kept in darkness at room temperature (24 °C) for 30 min. The working solution was prepared by dilution with a phosphate buffer (pH 7.2–7.4). Horseradish peroxidase (HRP) was then added to the diluted solution as the catalyst.

Sample reactivity was expressed by converting the fluorescence to a hydrogen peroxide (H_2_O_2_) concentration using the H_2_O_2_ calibration curve. After final dilution by adding DCFH-HRP working solution, six different H_2_O_2_ standards were prepared with concentrations of 1.0, 2.0, 3.0, 4.0, 5.0, and 10.0 × 10^−7 ^mol/L. A standard blank was obtained by adding the same amount of Milli-Q water to the H_2_O_2_ standard as to the fluorescent probe. The standards were incubated for 30 min at 37 °C immediately before testing.

To avoid impropriate light exposure to the sensitive fluorescent dye, all handling steps were done in darkness under a darkroom lamp emitting outside the excitation range of the dye.

### Chemical test

Chemicals and concentrations used in previous studies to prepare DCFH working solution for the detection of ROS are shown in Table 1. Cross comparisons were made to evaluate their performances.

**Table 1:** List of chemicals and concentrations used in previous studies

Media	Choices
Buffer	K Phosphate buffer and Na Phosphate buffer
Solvent	Methanol and Ethanol
Reactant	DCFH (2 µM), DCFH (5 µM) and DCFH (10 µM)
Catalyst	HRP (0.5 units/mL), HRP (2.2 units/mL) and HRP (3 units/mL)

### Sonication test for dispersing particles

Two types of particles were tested: FW2 and Aerosil 200. FW2 is a widely used type of black carbon NP which has been found to be chemically reactive (Sauvain et al. 2012). Aerosil 200 is a type of amorphous silicon dioxide. It is commercially available from Evonik Industries. Both FW2 and Aerosil 200 are widely applied in the nano field and have been well characterized and studied by both the manufacturer and many published authors (Bhowmick et al. 2010; Moritz and Nagy 2002; Kongsinlark et al. 2013; Sauvain et al. 2012, 2008). Moreover, they have been used in our home institute for several years and characterized by our colleagues, finding that they matched the information provide from the manufacturers. As a further reference, we performed TEM imaging which were included in supplemental data. These two particles were considered as the proxies for reactive and non-reactive NPs, respectively. Particle samples with the same concentrations were sonicated in sodium phosphate buffer (25 mM), DCFH-HRP working solution (5 µM), and Tween-80 (0.6 mg/mL) for 15 min to disperse them. Sonication was carried out in an ultrasonic water bath (Branson 5210, 2.8L, 180W) kept at a constant 37 °C. Sonication blanks were prepared by sonicating the reagent without particle samples.

### Fluorescence measurement

A 96-well multiple plate reader (Infinite M200, TECAN) was used to measure the fluorescent intensity (excitation wavelength 485 nm; emission wavelength 530 nm). The plate reader was kept at a constant 37 °C. The fluorescent signal was measured every minute 30 or 60 min.

### Determination of suitable sample concentrations

A wide range of FW2 NP concentrations were treated using the protocol developed in the present study. Also, alpha-Fe_2_O_3_ (hematite; 3310DX, SkySpring Nanomaterials Inc.) at NP and fine particle (hematite; I-1039, Chemco) scales were tested at high concentrations—in the mg/mL range. Some samples showed an unexpectedly low signal and two approaches were used to examine why this might be, namely adding additional HRP, and ultrafiltration to remove the particles. Ultrafiltration tubes (Vivaspin 15R, Sartorius Stedim Biotech) were used and centrifuged (SW9RH, Firlabo) at 400 rpm for 10 min. To further support our hypothesis, absorption spectra were obtained by measuring the absorbance of the samples from wavelengths of 230 to 900 nm using the 96-well multiple plate reader.

The fluorescent working solution with HRP was always consumed within 3 days to minimize the possible interference of having a relatively big background.

## Results

### Sodium phosphate buffer versus potassium phosphate buffer

**Fig. 1:**
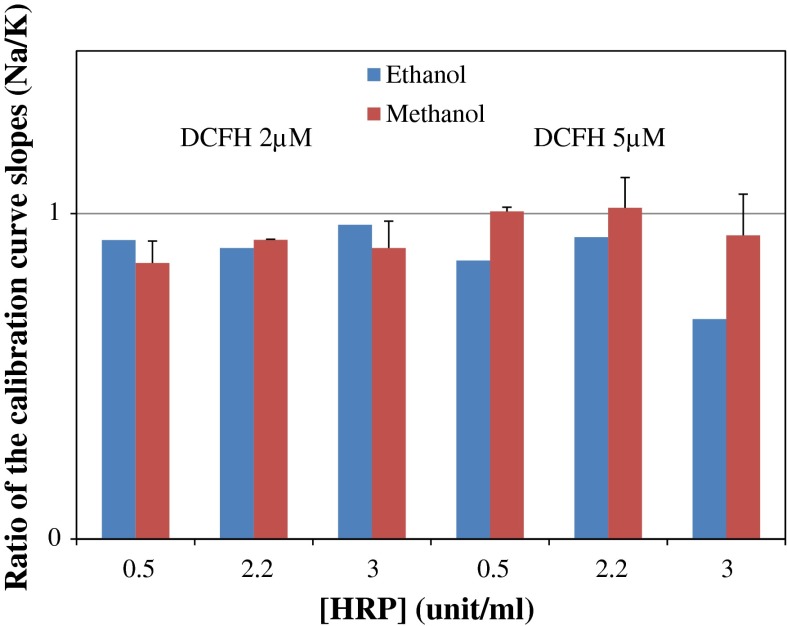
Performance comparison of Na buffer *vs* K buffer: calibration curve slopes

The ratios of the calibration curve slopes obtained from testing Na buffer against K buffer with DCFH-DA powder dissolved in ethanol and methanol were close to 1 for all the concentrations tested (Fig. 1). Since the slope of the H_2_O_2_ calibration curve is an essential factor for calculating the reactivity, the comparison of the slopes can inform us about the relative performance of the two buffers.

### Methanol versus ethanol as the stock solution solvent

In general, the R^2^ obtained from the calibration curves, using either methanol or ethanol as the DCFH stock solution reagent, were relatively high (>0.8) (Table 2). Similar slopes and R^2^ were observed using both reagents, with slightly better correlations when using ethanol.

**Table 2:** Performance of methanol *vs* ethanol: *R* ^2^ comparison

	Methanol	Ethanol
K buffer (2 µM DCFH)		
HRP 0.5 unit/mL	0.9435	0.9457
HRP 2.2 unit/mL	0.9209	0.9759
HRP 3 unit/mL	0.9299	0.9641
Na buffer (2 µM DCFH)		
HRP 0.5 unit/mL	0.8126	0.9343
HRP 2.2 unit/mL	0.8867	0.9784
HRP 3 unit/mL	0.8986	0.9804

### Concentrations of reactant and catalyst

Cross comparisons were made using 2, 5, and 10 µM DCFH working solution with 0.5, 2.2, and 3 units per mL HRP. H_2_O_2_ was used as the standard. Similar curves were obtained using all three HRP concentrations indicating that HRP was not the limiting factor in the reaction. However, using higher HRP concentrations lead to bigger background noise. Also, with a greater amount of catalyst, the fluorescent working solution’s background reading tended to increase faster. We, therefore, selected a 0.5 unit/mL HRP for our standard methodology.

**Fig. 2:**
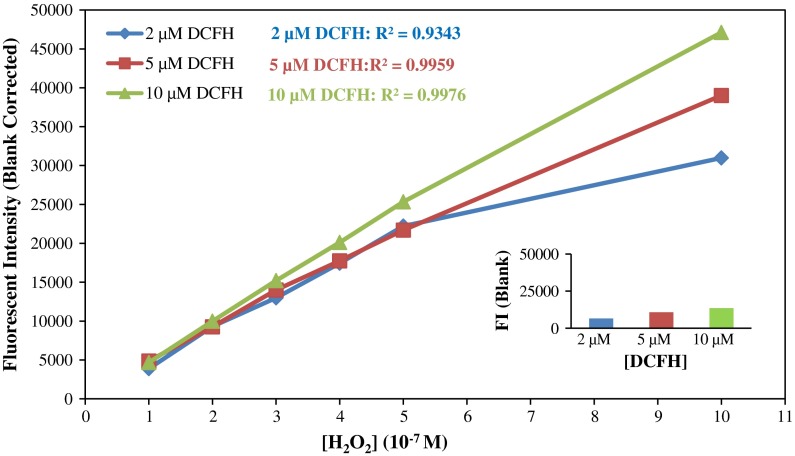
Performance comparison of different reactant concentrations with hydrogen peroxide

When using 0.5 unit/mL HRP, a smaller R^2^ was observed for 2 µM DCFH than for 5 µM or 10 µM (Fig. 2). Stable, linear calibration curves were obtained using both 5 µM and 10 µM DCFH. This indicated 2 µM DCFH was not sufficient for a relatively big generation of ROS, e.g., 10 × 10^−7 M H_2_O_2_. However, steeper slopes and bigger blanks were found using 10 µM DCFH.

**Fig. 3:**
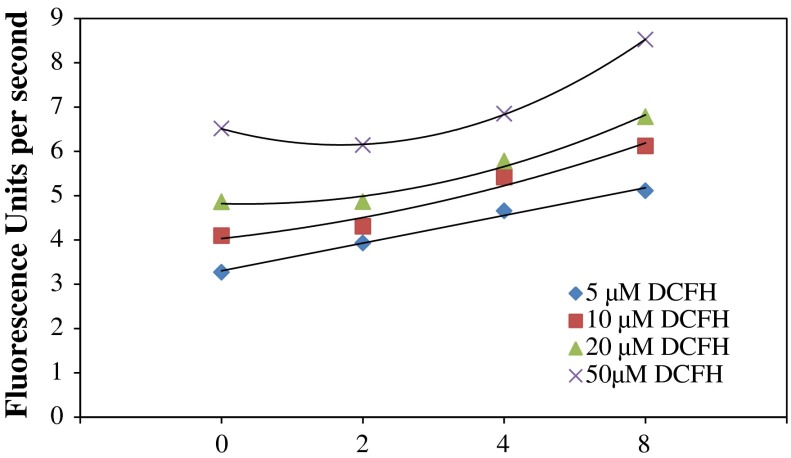
Exploration of suitable reactant concentration: slopes of the dynamic curves obtained by applying DCFH with FW2 NPs

Further, three FW2 NP samples at concentrations from 2 to 8 µg/mL were applied to 5, 10, 20, and 50 µM of reactant. Dynamic curves were developed based on their fluorescent intensity measurements taken every minute for 1 h. Figure 3 shows that the slopes obtained agreed with the observed phenomena when using H_2_O_2_: with 5 µM DCFH, we clearly distinguished the four NP sample concentrations. The background fluorescence using 5 µM was also the lowest out of the four reactant concentrations. Clearly, distinguishable slopes were observed when using different sample concentrations. Also, the 5 µM reactant slope showed a relatively linear increase as concentrations of the NP suspensions increased. A similar phenomenon was observed using 10 µM reactant, but there was higher noise and a binomial increase was found. This trend continued when the DCFH concentration was increased further. For 20 µM DCFH, it was already hard to tell the difference between blank and 2 µg/mL FW2. At 50 µM, the fluorescence dynamic curve showed a steeper slope with the blank reactant than with the low FW2 sample suspension.

The 5 µM DCFH with 0.5 unit/mL HRP working solution, sealed and covered with aluminum foil, could be stored in a refrigerator (at 4 °C) for at least a week. An increased blank was observed, but the level of increase was acceptable (30–50 % by the eighth day; raw data not shown).

### Comparison of sonication methods

**Fig. 4:**
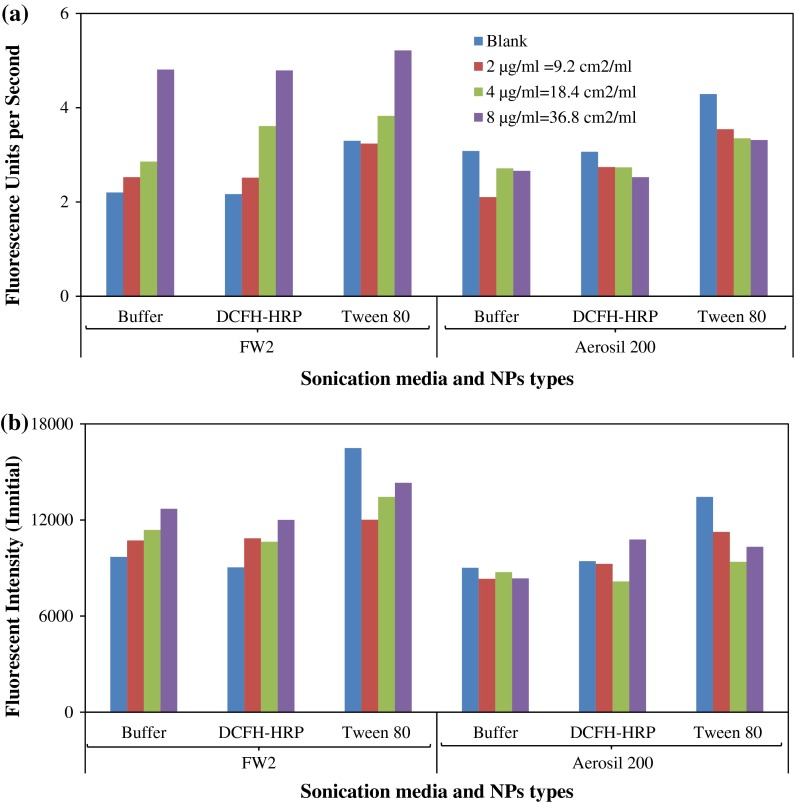
Exploration of different dispersing media (sonication with different NP types)

The most significant influence on the sample dispersion methods tested was the medium used to sonicate the samples. Figure 4 shows the comparison between three different sonication reagents. Comparing the slopes and the blanks, the DCFH-HRP working solution yielded the lowest blank level. Moreover, its fluorescent intensity increased in proportion to the increase in NP concentration for a reactive NP, in this case FW2. Tween-80 actually contributed to fluorescence as blank values were higher than the NP suspensions in concentrations that are not influenced by absorbance phenomena.

### Exploration of the suitable sample concentration range and assessment of potential optical interference when using high sample concentrations

**Fig. 5:**
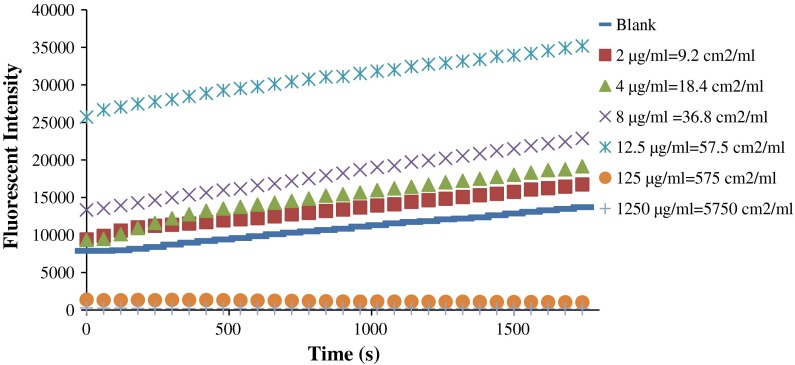
Suitable NP sample concentrations: DCFH assay response at several FW2 sample concentrations (2–1,250 µg/mL)

Our protocol was sensitive enough to reliably detect concentrations as low as 2 µg/mL in FW2 samples (Fig. 5). Units are presented in both mass per mL and surface area per mL. Greater fluorescent intensity was detected when the concentration was increased. However, the increase was not based on a linear relationship with the suspension concentration. A big increase in fluorescence was observed when the concentration was increased from 8 to 12.5 µg/mL. However, when the sample concentration was further increased to 125 µg/mL, we observed a significant decrease in fluorescence down to levels below the blank. A clearly reduced fluorescence with a signal lower than the blank was observed for all sample concentrations in the magnitude of mg/mL.

To test whether the high levels of NPs used up the catalyst and limited the reaction, much higher HRP concentrations were tested. No obvious changes were observed.

Another hypothesis was that the NPs settled down to the bottom of the wells (but remained in suspension) thus blocking the signal. This was investigated using ultrafiltration. After ultrafiltering each sample, the fluorescence of the remaining solution was tested again. The signal intensity was now close to the blank fluorescence. This approach was also tested with Fe_2_O_3_ NPs and fine particles, and similar results were obtained.

**Fig. 6:**
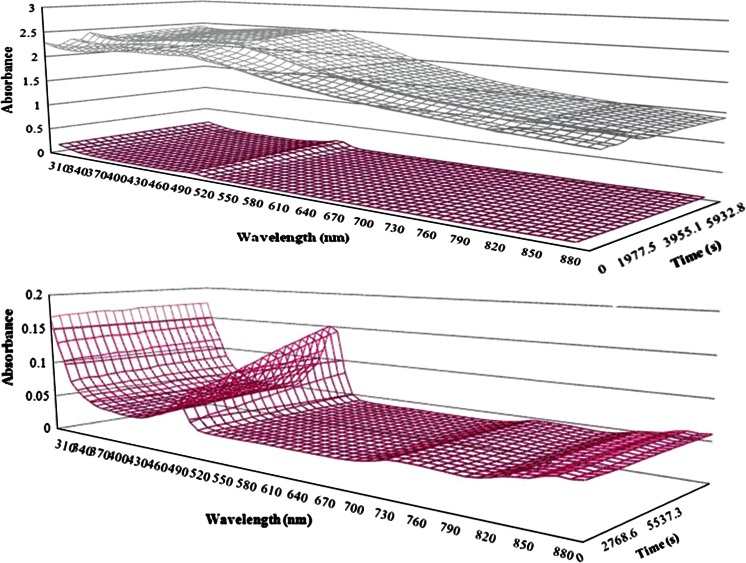
Absorbance test and comparison of pure DCFH working solution (*magenta*) and NP suspension (*gray*): **a** Comparison of the two readings; **b** same graph of pure DCFH, but with enlarged scale

For a fluorescent solution without particle samples, the absorbance was generally relatively low (Fig. 6a). A high absorbance at low wavelengths (<350 nm) was expected from the absorbance of the plastic well plate. A clear peak was observed in the range of 485 nm to 530 nm, which was the range of the fluorescent signal. However, the peak was negligible compared to the absorbance from the high particle concentration suspension (Fig. 6b). Across the entire wavelength spectrum measured, the 1.25 mg/mL Fe_2_O_3_ NP suspension yielded approximately ten times higher absorbance. A peak was again observed in the 485–530 nm range, implying the absorbance issue could be more severe in this range. We also tested the absorbance using a 12.5 mg/mL Fe NP suspension. As expected, the signal was too high to be measured.

## Discussion

**Fig. 7:**
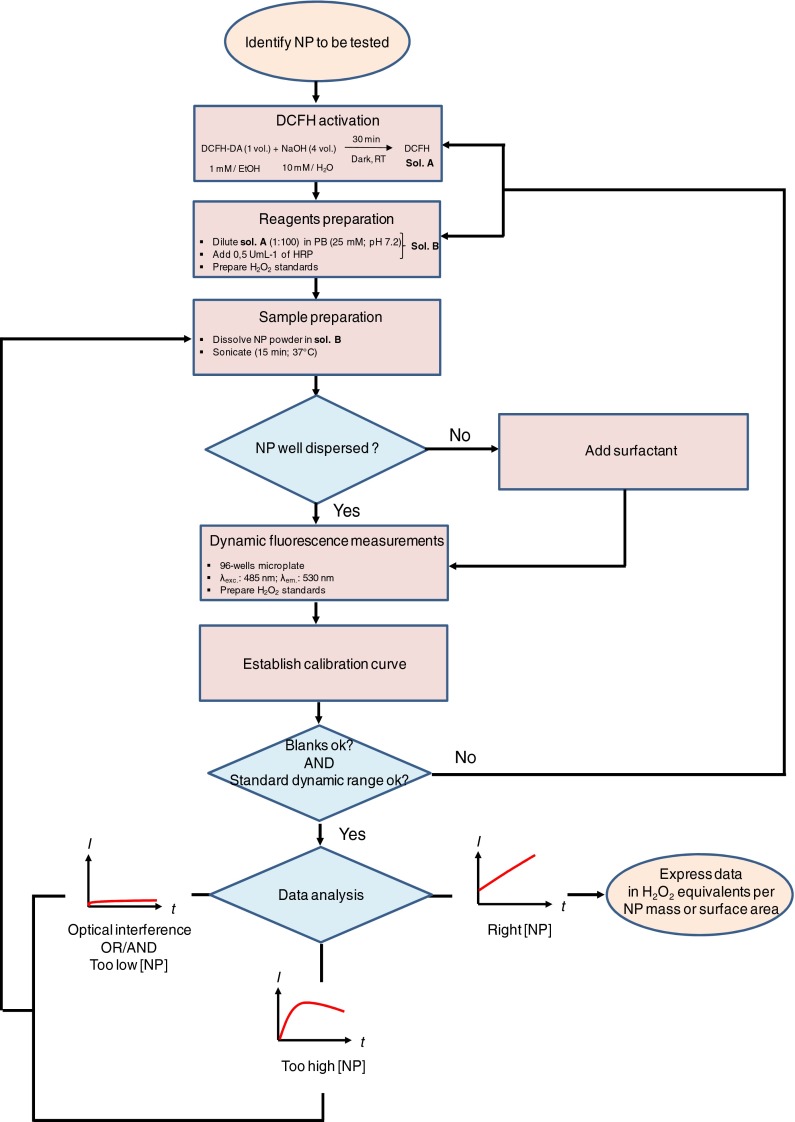
General flow chart of the proposed assay with decision logic

This study provides further evidence that, when applied appropriately, the DCFH assay is a valid and reliable NP reactivity test. The assessment of the influence of the different reactants on the test results provides an understanding of previously reported conflicting results (Sauvain et al. 2012; Pal et al. 2012). The refinement of the DCFH-method proposed here should help avoid such issues in the future. Figure 7 shows the proposed assay in a flow chart.

### Chemicals chosen to prepare the fluorescent reactant

The rates of generating ROS for the same H_2_O_2_ standard were comparable—close to 1—for both the sodium and potassium buffers; they, thus, showed similar performance. It seems that they both provided suitable conditions for the reaction between the fluorescent probe and ROS. However, the sodium buffer is more commonly used, especially in cell research media. To make the acellular protocol more compatible to cellular tests, which usually need to avoid high potassium concentrations, we propose using the sodium phosphate buffer.

We also obtained very similar results when using methanol or ethanol as the dissolving solvent for DCFH_2_-DA powder. The raw data showed a slightly stronger response when using methanol. Previous research (Jiang et al. 1990; Wolff 1994) suggested that alcohol compounds such as methanol and ethanol could generate free radicals when used in a ferrous oxidation method. Thus, it is possible that methanol was contributing to the production of radicals when used in the DCFH test. However, the difference was small and the possible interference from using the solvent could be normalized with reference to the sampling blank. The main reason we prefer ethanol over methanol is that it is less toxic and more compatible with solutions used in cell research. Furthermore, at the highest concentrations (see Fig. 1), methanol seemed to have slightly influenced the reactions.

### Suitable reactant concentrations

The DCFH fluorescent probe is sensitive to light and can be oxidized in ambient air (Chen et al. 2010). The more concentrated the reactant is, the bigger initial background it has. Also, the increase of this already relatively high background is faster than less concentrated reactants. Thus, the major concern when applying high fluorescent probe concentrations (such as 10 µM) is that more auto-oxidation could lead to a high background, which may in turn reduce the accuracy of the detection of low ROS generation. Furthermore, it is very likely that a highly concentrated reactant solution cannot be stored as long as lower concentrations. On the other hand, a low concentration reactant such as 2 µM DCFH may not be concentrated enough to detect high ROS content. Thus, a 5-µM DCFH working solution is generally recommended, while a 2-µM solution may be applied when the expected capacity for the generation of ROS is rather low (Fig. 2).

### Preferred dispersing reagent

When using the DCFH fluorescent method for testing NPs, the particle samples should be dispersed in solutions first. Many studies used sonication and its value has been attested (Zhao and Hopke 2011; Sauvain et al. 2012; Pal et al. 2012). There has been disagreement on which media should be used to prepare particle suspensions and many efforts to evaluate different ones.

Both Foucaud et al. (2007) and the present study have demonstrated that adding different media can change the baselines for DCFH oxidation. It seems that the dispersion media become involved in the reactions and contribute to the generation of radicals. Thus, evaluation methods should not only consider the effectiveness of NP dispersions, but also the possible interference when using these media. Moreover, strategies for dealing with different NPs should be adapted to their hydrophilic or hydrophobic nature.

Many aerosol-related studies (Hung and Wang 2001; Venkatachari et al. 2005; Zhao and Hopke 2011) directly used DCFH-HRP working solution as the medium for sonicating particle samples and achieved reasonable results. Our data showed that this approach has the advantages of a relatively clean background and having the sensitivity to properly distinguish different sample concentrations. Also, it is easy to carry out, without potential interference from surfactants. Dissolving NP samples directly into the fluorescent working solution could avoid possible sample loss during sonication, therefore, this would be preferable. We would also recommend verifying whether special media have to be added based on the NP sample’s properties. In the latter case, attention should be paid to the level of background noise. Moreover, since quite different results were obtained using different sonication protocols, the use of a standard chemical, such as H_2_O_2_, is strongly recommended so as to provide a reference against which the generation of ROS can be measured.

Sager et al. (2007) applied black carbon and titanium dioxide NPs to three media, namely phosphate buffered saline (PBS), rat, and mouse bronchoalveolar lavage fluid and PBS with dipalmitoyl phosphatidylcholine (DPPC) and/or mouse serum albumin (Sager et al. 2007). They concluded that buffer sonication was not satisfactory, which agrees with our results. Their data also showed BAFL and PBS with protein and DPPC, performed well. Foucaud et al. (2007) also investigated the performances of different media by using light microscopy (Foucaud et al. 2007). Analysing the optical microscope images of 300 µg/mL stock solution, they found that a medium containing NaCl saline with 1 % bovine serum albumin (BSA) and 0.025 % DPPC, or NaCl with BSA only, was superior to NaCl or NaCl with DPPC only. This was because these combinations with DPPC produced less agglomeration after 10 min of sonication. Also, after adding BSA, no NP agglomeration, deposition, or settling was observed after leaving the 30 µg/mL diluted solutions undisturbed for 30 min.

### NPs’ high potential for generating ROS and their larger surface area

The present study observed a very high fluorescent signal in a 57.5 cm^2^/mL suspension of FW2 NPs. Similarly, both Wilson et al. (2002) and Foucaud et al. (2007) reported that the maximum fluorescent intensity achieved was at a concentration of about 30 µg/mL of carbon black NPs, which corresponds to about 76 cm^2^/mL of suspension. Similar surface dose-dependent results were observed by Sun et al. (2011) when testing various metal oxide NPs (MgO, CuO, and ZnO). Therefore, for reactive-type NPs, it is possible that their capacity to generate ROS could be inferred based upon their surface area. This capacity would increase until surface saturation. When we increase the sample concentration still further—past the point of saturation—the extra particle mass will quench the fluorescent signal and the observed fluorescence will decrease.

By comparing the reactivity of ultrafine and fine carbon black samples, Wilson et al. (2002) illustrated that the much higher reactivity detected from ultrafine samples could be attributed to their proportionally much bigger surface areas. Surface function is essential to the study of toxicity because it is at this interface that reactions happen. A major mechanism for the generation of ROS by particles was the surface properties participation in redox cycles (Nel et al. 2006). It has been noted that surface area is related to the capacity for generating ROS (Koike and Kobayashi 2006). Thus, using surface area as the dose unit may be a better approach for investigating and reporting on the generation of ROS.

### Suitable NP sample concentrations for the analysis of their capacity to generate ROS

Sample concentrations should be chosen based on a study’s particular aim. For example, relatively low concentrations should be used to simulate an ambient situation, but overly low concentrations might not be suitable since there may not be enough samples to generate detectable amount of ROS. High NP sample concentrations should be used when aiming to simulate an environment with a high expected content of ROS, but caution should be given when analyzing these concentrations (Fig. 5).

However, to characterize the reactivity of a specific type of NP, a reasonable range of NP concentrations should be chosen and evaluated. The most likely reason for the decrease in fluorescence at high particle concentrations seems to be optical interference. Our attempt to add additional HRP to the highly concentrated particle suspension indicated that the amount of HRP was not responsible for the extra-low fluorescence. We hypothesized that, in fact, the high level of particles present in the plate well caused them to be deposited at the bottom of the well due simply to gravity. This deposition further blocked the fluorescent signal coming from the bottom. This hypothesis was supported by the opacity observed when using high concentrations of carbon black NPs (Foucaud et al. 2007). Wilson et al. (2002) also reported decreased fluorescence because of absorbance when using fine particle carbon black samples (Wilson et al. 2002). Furthermore, our absorbance scans using 1.25 mg/mL Fe_2_O_3_ NPs confirmed that big sample concentrations could lead to absorbance issues. This high absorbance level could lead to serious detection problems. From our ultrafiltration data on different iron particle samples, it seems too much particle mass could lead to an absorbance issue for both fine-scale and nano-scale particles. This would also be expected for even bigger particulate matter. Another possible reason could be that the free DCFH adsorbs to the NP surfaces and is removed from the suspension, reducing the effectiveness of the fluorescent probe. A clear dose-dependent decrease was also shown when using Fe_3_O_4_ NPs (Doak et al. 2009). It was suspected this was because the fluorescent signal was absorbed on the NP surfaces (Pal et al. 2012).

It is worth pointing out that although high sample concentrations can cause measurement interferences for almost all NPs, the bias effect level also depends on the NPs’ properties. For example, the ability to absorb and scatter light can greatly influence optical interference. Also, NPs with a relatively high density can demonstrate faster sedimentation than less dense NPs. In our study, a sample concentration of 125 µg/mL caused interference for both FW2 and Fe_2_O_3_ NPs. However, at the beginning of the FW2 analysis, the fluorescent signal was already 87 % lower than that of the blank sample, and after half an hour it was down to 95 % lower. In comparison, the Fe_2_O_3_ signal was 70 % lower than the blank at the start and 74 % lower after half an hour.

### Possible reasons for the conflicting results reported previously

Although several previous studies used similar DCFH approaches on the same types of NPs, their conclusions varied, even to the extent of being contradictory (Pal et al. 2012; Sauvain et al. 2012). However, their different experimental approaches and conditions could in fact help to clarify certain disagreements in their studies of NP reactivity. For example, Sauvain et al. (2012) evaluated three acellular reactivity tests, namely the DCFH assay, the DTT assay, and the ascorbic acid assay, and reported a relatively high oxidation potential for Ag NPs (Sauvain et al. 2012). However, Pal et al. (2012) found that Ag NPs were not reactive after 1 wt% BSA/0.9 wt% NaCl cup sonication and 0.7 wt% Triton-X 100 probe sonication (Pal et al. 2012). The sonication media can of course affect the conclusion, yet Pal et al. (2012) used sample suspension concentrations ranging from 0.1 to 0.5 mg/mL. It is possible that these relatively high particle concentrations caused optical interference that impeded the detection of ROS generation. Our data for FW2 showed that sample concentrations in the magnitude of µg/mL would be suitable for the purpose of reactivity studies. Therefore, to suitably characterize the reactivity of NPs, appropriate sample suspension ranges should be explored and applied based on the NPs’ physical and chemical properties and surface area information. In other words, questions about whether NPs might be a potential source of ROS generation should be answered based on a situation involving a specific NP concentration, rather than generally.

## Conclusions

We have designed a sensitive, feasible, and reliable protocol for characterizing NP reactivity using the popular DCFH fluorescent probe. We evaluated different variations of the DCFH assay and have provided a unified approach that should allow appropriate assays to be successfully carried out. We were also able to clarify the possible reasons for conflicting conclusions reported in the past.

In order to produce reliable and comparable reactivity data, attention should be given to the chemicals chosen. To be more compatible with cell culture studies, but also for reasons of laboratory safety, we suggest using chemicals with low toxicity: in our case, sodium phosphate buffer and ethanol. Overly high reactant concentrations may prevent researchers from seeing the reaction itself, especially when a low sample concentration is used; overly low concentrations may not yield appropriately high signals. Sonication media may take part in the reaction and this potential interference must be checked for. Our results suggest that expressing NP reactivity as a function of the surface area helps in deciding on a reasonable sample suspension range, which is essential for accurate measurement. Possible saturation and optical interference should also be evaluated.

We propose that the protocol developed here be further adapted into a standard for studying the capacity of NPs to generate ROS. It should be noted that, based on specific study aims and the NPs involved, adapting the standard may be required, yet this could provide the basis of a decision tree to guide researchers. As such, the data in this study could be a useful reference for decision making.

## Electronic supplementary material

Below is the link to the electronic supplementary material.

Supplementary material 1 (DOCX 355 kb)
