# Supplementary material for: Detecting the oxidative reactivity of nanoparticles: a new protocol for reducing artifacts
Source: J Nanopart Res. 2014 Jun 28;16(7):2493. doi: 10.1007/s11051-014-2493-0 (PMC4092240; doi:10.1007/s11051-014-2493-0)
Supplement: Supplementary file 1 — Supplementary material 1 (DOCX 355 kb) [file 11051_2014_2493_MOESM1_ESM.docx]

Supplemental Data to ‘Detecting the oxidative reactivity of nanoparticles: a new protocol for reducing artifacts’

Jiayuan Zhao, Michael Riediker

Institute for Work and Health, University of Lausanne, Rte de la Corniche 2, CH-1066 Epalinges – Lausanne, Switzerland.







Figure 1. TEM images with nanoparticle concentrations at 12.5 µg/ml (scale bar: 200nm): up: FW2; down: Aerosil 200.
